# Estimation of step-by-step spatio-temporal parameters of normal and impaired gait using shank-mounted magneto-inertial sensors: application to elderly, hemiparetic, parkinsonian and choreic gait

**DOI:** 10.1186/1743-0003-11-152

**Published:** 2014-11-11

**Authors:** Diana Trojaniello, Andrea Cereatti, Elisa Pelosin, Laura Avanzino, Anat Mirelman, Jeffrey M Hausdorff, Ugo Della Croce

**Affiliations:** Information Engineering Unit, Department of Political Sciences, Communication Sciences and Information Engineering (POLCOMING), University of Sassari, V.le Mancini 5, Sassari, 07100 Italy; Interuniversity Centre of Bioengineering of the Human Neuromusculoskeletal System, Sassari, Italy; Department of Neuroscience, Rehabilitation, Ophthalmology, Genetics and Maternal Child Health, University of Genova, Genova, Italy; Department of Experimental Medicine, Section of Human Physiology and Centro Polifunzionale di Scienze Motorie, University of Genova, University of Genova, Genova, Italy; Center for the study of Movement, Cognition and Mobility, Department of Neurology, Tel Aviv Sourasky Medical Center, Tel Aviv, 64239 Israel; Department of Physical Therapy, Sackler School of Medicine and Sagol School of Neuroscience, Tel Aviv University, Tel Aviv, Israel

## Abstract

**Background:**

The step-by-step determination of the spatio-temporal parameters of gait is clinically relevant since it provides an estimation of the variability of specific gait patterns associated with frequent geriatric syndromes. In recent years, several methods, based on the use of magneto-inertial units (MIMUs), have been developed for the step-by-step estimation of the gait temporal parameters. However, most of them were applied to the gait of healthy subjects and/or of a single pathologic population. Moreover, spatial parameters in pathologic populations have been rarely estimated step-by-step using MIMUs. The validity of clinically suitable MIMU-based methods for the estimation of spatio-temporal parameters is therefore still an open issue. The aim of this study was to propose and validate a method for the determination of both temporal and spatial parameters that could be applied to normal and heavily compromised gait patterns.

**Methods:**

Two MIMUs were attached above each subject’s ankles. An instrumented gait mat was used as gold standard. Gait data were acquired from ten hemiparetic subjects, ten choreic subjects, ten subjects with Parkinson’s disease and ten healthy older adults walking at two different gait speeds. The method detects gait events (GEs) taking advantage of the cyclic nature of gait and exploiting some lower limb invariant kinematic characteristics. A combination of a MIMU axes realignment along the direction of progression and of an optimally filtered direct and reverse integration is used to determine the stride length.

**Results:**

Over the 4,514 gait cycles analyzed, neither missed nor extra GEs were generated. The errors in identifying both initial and final contact at comfortable speed ranged between 0 and 11 ms for the different groups analyzed. The stride length was estimated for all subjects with less than 3% error.

**Conclusions:**

The proposed method is apparently extremely robust since gait speed did not substantially affect its performance and both missed and extra GEs were avoided. The spatio-temporal parameters estimates showed smaller errors than those reported in previous studies and a similar level of precision and accuracy for both healthy and pathologic gait patterns. The combination of robustness, precision and accuracy suggests that the proposed method is suitable for routine clinical use.

**Electronic supplementary material:**

The online version of this article (doi:10.1186/1743-0003-11-152) contains supplementary material, which is available to authorized users.

## Background

Walking allows humans to move forward by alternatively and repetitively swinging their left and right lower limbs. The gait pattern can be segmented into cycles that are typically divided into different phases in relation to the position of each foot with respect to the ground and of one foot with respect to the other (e.g. stance, swing and double support phases). The duration of the gait cycle phases is estimated by identifying the initial (IC) and final foot contacts (FC) timings, usually referred to as gait events (GE). The duration of the gait cycle is typically estimated by determining the time interval between two consecutive ICs of the same foot. The distance, along the direction of progression, traversed during a gait cycle, is referred to as stride length. Both stride length and duration can be seen as the sum of two consecutive steps, i.e. the distance traversed or the time interval between an IC and the following one of the contralateral limb [1].

From a lower limb kinematics perspective, human walking requires that: a) the two lower limbs alternate their swing phase while the opposite foot is in contact with the ground; b) at some point in stance there is at least one foot point fixed with respect to the ground (i.e. no sliding), c) swing begins with a roto-translation of the shank and ends with foot impact with the ground. The above-mentioned requirements apply to both healthy and pathologic gait and therefore can be exploited to detect GEs and spatio-temporal parameters.

A step-by-step determination of the spatio-temporal parameters is of great clinical relevance [2–5]. Often, the variability of different aspects can provide information that is independent of the average values. Variability of gait parameters has been associated with frequent geriatric syndromes such as falls, dementia and frailty [6]. In addition, gait variability has been associated with fall risk and disease progression in patients with Parkinson’s disease [7, 8]. Variability is also larger in patients with other movement disorders, like Huntington’s disease and in post-stroke patients. Because variability reflects the step-to-step consistency of the gait, it has been used to describe the quality of the gait pattern and dynamic stability.

Various sensing technologies have been proposed to estimate step-by-step gait temporal and spatial parameters. Force platforms, instrumented mats, and footswitches are examples of devices sensing the contact of the foot with the ground. Motion analysis systems and magnetic and inertial measurement units (MIMU) as well as combinations of MIMUs and other wearable technologies (i.e. pressure sensors [9]) have also been used to estimate GE timings from body segment motion [10, 11]. To some degree, force platforms and instrumented mats suffer from the same limitations. They require extensive laboratory space, force subjects to walk in a specific environment and are relatively costly. Their main advantage is the possibility of estimating spatial gait parameters in addition to temporal parameters. Foot switches are portable and relatively inexpensive but may require extensive subject set up and can provide temporal parameters only. Motion capture systems capabilities go beyond the estimation of the gait spatio-temporal parameters, since they are devised for 3D point kinematics measurements. These systems are pricier than the above mentioned alternatives and generally can only capture a small number of consecutive steps.

The use of the MIMUs has been increasingly explored in the recent years thanks to the development of miniaturized sensing technology and the consequent improved wearability. However, MIMU-based recordings require appropriate processing to estimate gait parameters for clinical applications [12].

A number of authors have proposed methods applied to MIMU measurements for estimating gait temporal parameters [13–23] or spatio-temporal parameters [24–32]. A single sensor placed on the lower trunk has been proposed for healthy subjects [33–37] and pathologic gait [38–42]. A larger number of methods have been proposed using MIMUs attached to the lower limbs: on the feet or shoes [19, 23, 25, 26], on the shanks [13–16, 27], thighs [21], or both shanks and thighs [18, 24]. In general, the farther from the contact point the MIMU is placed, the more difficult the GEs identification is. However, placing the MIMUs on the shanks may offer some advantages over the feet (or shoes). In fact, the shank is a more rigid segment and may allow for a firmer attachment of the MIMU [13]. Moreover, the recorded signals were found to be less variable across homogeneous subjects populations when MIMUs are mounted on the shank than when mounted on the foot [43].

When the MIMU is attached to a lower limb segment, the GEs detection and the determination of gait cycle phases is often based on the analysis of the sagittal angular velocity features [13, 14, 23, 26, 27] or, less frequently, of the acceleration features [15, 17, 19, 20], applying approaches such as empirically determined thresholds [13, 26, 27], frequency analysis [24] and machine learning algorithms [23].

Methods for the determination of stride length from MIMU signals have also been proposed either using abstraction models, human gait models or signal integration [44]. Methods based on abstraction models perform poorly since the accuracy of the spatial parameters estimation depends on the completeness of training data; difficulties in controlling the performance across subjects have been also reported. The use of predefined human gait models requires subject specific anthropometric measurements. Since such models are based on the observation of physiological gait, accuracy issues in applying them to pathological gait patterns have been reported [44]. The signal integration methods consist of obtaining linear displacements by double integrating the MIMU gravity-compensated linear acceleration in the global reference frame [31, 37]. However, due to the presence of drift in acceleration signals [45], the inaccuracy related to the estimation of the MIMU orientation [46] and the value of the constant of integration of the relevant signals (initial condition) [47], the estimate of gait spatial parameters is extremely poor unless some countermeasures are implemented. The cyclical nature of gait is typically used to reduce the detrimental effects of the drift by restricting the interval of integration time to the duration of a single gait cycle [47]. This requires the identification within the cycle of an instant of known velocity to be used as the initial value in the integration of the acceleration. The zero velocity update (ZUPT) is generally applied for this purpose to foot mounted MIMUs at the instant of flat foot [26, 28, 48]; when the MIMU is mounted on the shank, an inverted pendulum model is often used to estimate the initial integration value [30]. In addition, some de-drifting functions have been proposed [25, 26, 28]. The above mentioned expedients rely heavily on the quality of GE estimates. In fact, errors in determining the gait cycle and the instants of minimum velocity, as well as the chosen de-drifting function could compromise the estimate of gait spatial parameters.

Most of the studies mentioned above validated the proposed GE detection methods on healthy subjects [13, 14, 16, 19, 23, 26]. The validity of MIMU based methods for the estimation of gait spatio-temporal parameters in clinical applications is still an open issue. Some studies applied the proposed method to the gait of elderly [24, 28], spinal cord injuried [17], Parkinsonian [15, 27, 32], amputee [22] or patient’s with prostheses [49]. Spatial parameters in pathologic gait have been estimated mostly as average values and only in a few studies on a step-by-step basis [24, 26–29, 32, 50]. Only a few of the above mentioned studies have been validated against a gold standard. In a recent study, Yang et al. [24] reported that methods for the determination of the gait cycle phases failed when the deviations of the angular velocity patterns from those typical of normal gait are not negligible. Such deviations are often due to impairments and consequent compensatory strategies. For example, hemiparetic gait is often characterized by an increased lateral displacement of the foot during swing in the paretic limb, consistently with limb vaulting to further assist limb clearance [5]. Other gait abnormalities, such as choreiform gait, also known as "drunken gait", are characterized by staggering from side to side, with lateral swaying, and stride-by-stride lateral deviations from forward direction during walking [51], while Parkinsonian gait is generally characterized by small shuffling steps and a general slowness of movement [3]. Each of the abnormal gait patterns reported above affects the MIMU signal patterns. Therefore a highly reliable method for the step-by-step estimation of spatio-temporal parameters should be validated for both healthy and heavily impaired gait.

The aim of this study was to propose and validate a method, based on the use of two MIMUs attached above the malleoli, for the determination of both temporal and spatial parameters that could reliably be applied to both healthy and heavily compromised gait. The above mentioned invariant characteristics of the lower limb kinematics characterizing human walking were exploited in developing the algorithm for the detection of the GEs instances, with the aim of enhancing its robustness across a variety of walking patterns by limiting the risk of experiencing extra and missed GEs. The GEs are detected by first identifying time intervals in which they cannot occur due to the intrinsic kinematic constraints, and then searching for GEs in the remaining portions of the gait cycle. The spatial parameters are determined by applying a modified version of a method originally developed for a waist-mounted MIMU [37]. Spatial and temporal parameters estimates were validated against those obtained using an instrumented mat.

## Methods

### Instrumentation

**Figure 1:**
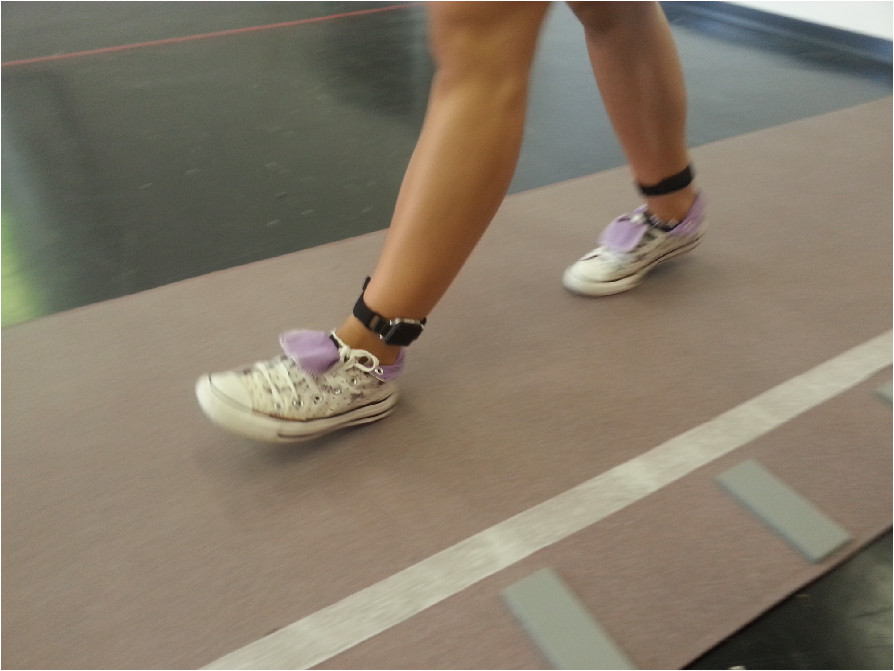
**Subject wearing two MIMUs attached above the ankles and walking on the instrumented mat used as gold standard for MIMU based estimates of gait spatio-temporal parameters.**

Two MIMUs (Opal, APDM) featuring a tri-axial accelerometer, a tri-axial gyroscope and a tri-axial magnetometer (unit weight 22 g, unit size 48.5 mm × 36.5 mm × 13.5 mm) were used. Sampling frequency was set at 128 Hz and accelerometer range at ±6 g. MIMUs were attached to the subject ankles (about 20 mm above the malleolus) with X, Y and Z axes pointing downward, forward and to the right, respectively (Figure 1). The physical quantities (proper accelerations, angular velocities and magnetic field vector) are measured with respect to the axes of a local frame aligned to the edges of the unit housing. An estimate of the MIMU local coordinate system (LCS) orientation with respect to the global coordinate system (GCS) was provided by the APDM proprietary software. A spot check of the MIMU performance was performed according to the guidelines proposed by [46].A gait pressure mat (GAITRite Electronic Walkway, CIR System Inc) acquiring at 120 Hz (spatial resolution accuracy: ±12.7 mm; temporal accuracy: ±1 sample) was used for validation purposes (Figure 1). The instrumented mat returned all GEs, temporal and spatial parameters under analysis. The MIMUs and the instrumented mat were synchronized (±1 sample).

### Subjects

Ten hemiparetic subjects (H), ten subjects with a choreic movement disorder (C), ten subjects with Parkinson’s disease (P) and ten healthy elderly (E) were enrolled from the out-patient Movement Disorders Clinic of the University of Genoa. Disease severity was determined by means of the Functional Ambulatory Category (FAC) [52] for the H subjects, the Unified Huntington’s Disease Rating Scale (UHDRS) [53] for the C subjects and the Unified Parkinson’s Disease Rating Scale (UPDRS) [54] for the P subjects. Demographic and clinical characteristics of the groups are summarized in Table 1. The Declaration of Helsinki was respected, all subjects provided informed written consent, and local ethic committee approval was obtained.

**Table 1:** **Summary of demographic characteristics and the clinical scores of the groups participating in the study (healthy elderly – E, hemiparetic – H, Parkinson’s disease – P and choreic – C)**

Subjects group	Gender	Age	Height	Weight	Clinical score
**E**	6 females	69.7 ± 5.8	161.8 ± 7.7	63.6 ± 5.7	-
	4 males				
**H**	2 females	58.6 ± 12.1	172.6 ± 5.8	82.5 ± 15.9	3.3 ± 1.5^(a)^
	8 males				
**P**	5 females	73.8 ± 5.7	166.1 ± 9.7	67.7 ± 9.3	62.7 ± 19.1^(b)^
	5 males				
**C**	5 females	50.3 ± 13.3	162.8 ± 5.1	60.6 ± 12.2	34.9 ± 16.9^(c)^
	5 males				

The clinical scores reported are: (a) FAC; (b) UPDRS; (c) UHDRS.

### Acquisition protocol

Subjects were asked to walk back and forth for about one minute along a 12-meter walkway with the instrumented mat placed two meters from the starting line where they stood with their feet together for a few seconds after the beginning of the MIMU acquisition. Subjects walked both at self-selected, comfortable speed (V1) and higher speed (V2). Subjects wore their own shoes and walking aids such as canes or tripods were allowed if used in daily life. Subjects could rest in between acquisitions if requested.

### Gait temporal and spatial parameter estimation

**Figure 2:**
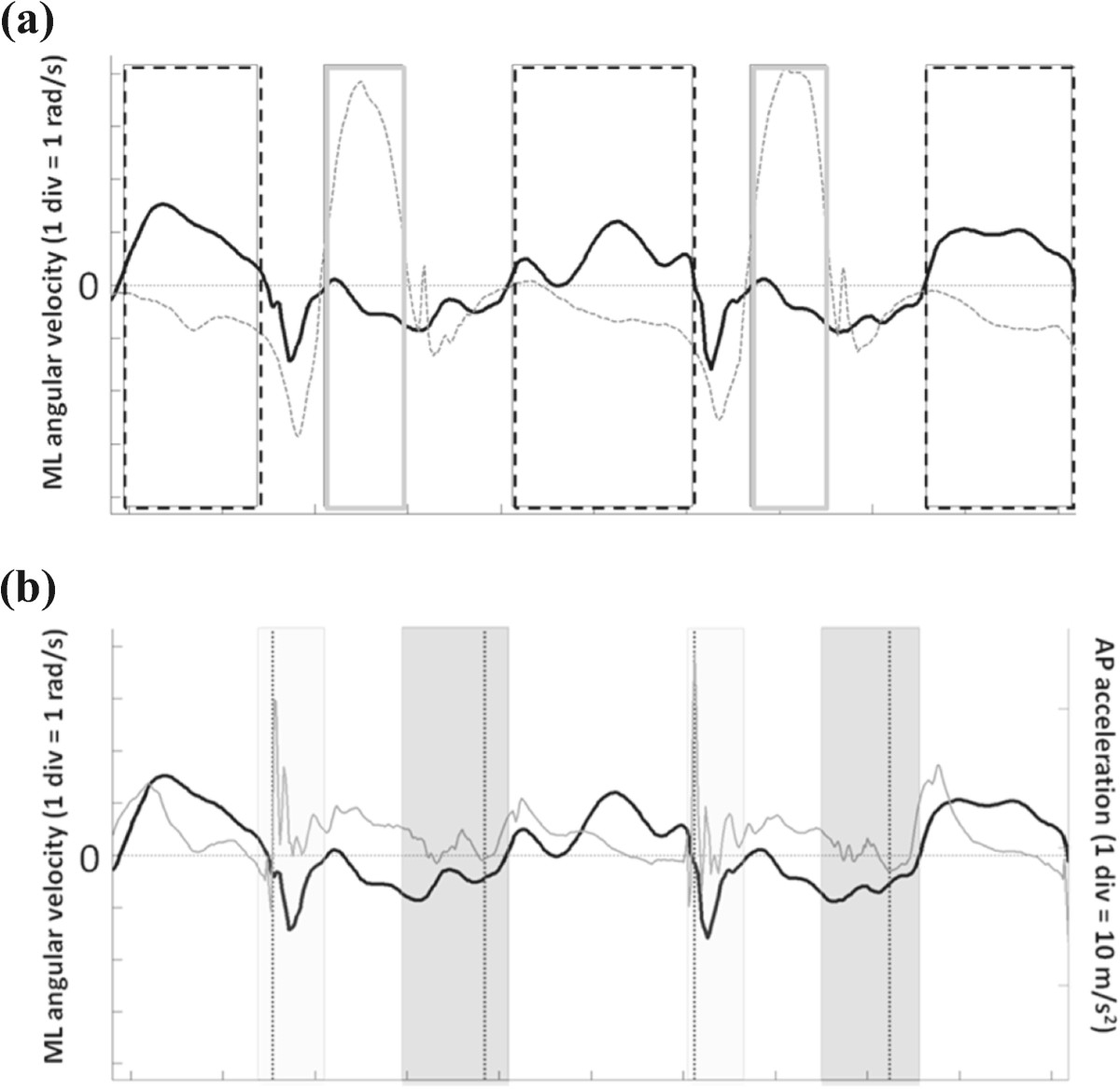
**Gait events detection from MIMU signals. (a)** Angular velocities in the sagittal plane (ω_z_) for a hemiparetic subject are reported (black line: affected side). Rectangular frames represent trusted swing (dotted line) and trusted stance (solid line) intervals for the affected limb. **(b)** ML angular velocity (black line) and AP acceleration (gray line) for the affected side of a hemiparetic subject. Colored boxes represent time intervals for the IC (light gray) and FC (intense gray) search; dotted vertical lines represent the GEs timings.

The algorithm implemented for detecting GEs required as first step the identification of time intervals in which no GE can occur (intervals of trusted swing - *T*_*SW*_). Their identification is based on the angular velocity signals in the sagittal plane (ω_z_) obtained from the gyroscopes. In fact, in both normal [26] and Parkinson’s disease gait [27], the ω_z_ recorded from either the shank or the foot shows the largest values at mid-swing and a *T*_*SW*_ can be defined as the time interval with ω_z_ larger than a set threshold (20%) of its local maximum value M_p_. If the ω_z_ crossed the threshold multiple times within a fraction of a second, as it occurs in some pathologic gait patterns, the *T*_*SW*_ was defined as the interval between the first and last threshold crossings including ML angular velocity local maxima (see Figure 2a). The following additional conditions also had to be satisfied: i. the minimum *T*_*SW*_ duration was set at 100 ms; ii. two consecutive *T*_*SW*_ of the same foot were separated by a minimum of 200 ms.

Since the two lower limbs alternate their swing phase while the opposite foot is in contact with the ground, the *T*_*SW*_ of a lower limb can be used as interval of trusted stance (*T*_*ST*_) of the other limb. Therefore, when coupled, the two *T*_*SW*_ allow for the identification of both *T*_*ST*_ and *T*_*SW*_ for each lower limb, reducing considerably the size of the time intervals in which ICs (*T*_*IC*_) and FCs (*T*_*FC*_) have to be searched, and consequently the risk of detecting extra GEs (see Figure 2b for details). The IC was identified as the minimum value of the ML angular velocity [26, 27] occurring in *T*_*IC*_ before the instant of maximum AP acceleration. The FC was identified as the instant of minimum AP acceleration in the *T*_*FC*_, since it is expected to occur at the time of a sudden motion of the shank preceding the instant of the last maximum AP acceleration value in *T*_*FC*_ (Figure 2b). Missed GEs could therefore occur only if *T*_*SW*_ were missed, which could happen only if the subject’s feet progressed without swinging. Once the IC and FC were determined for each gait cycle, stride, step, swing and stance times were computed for both sides.

The stride length was estimated as the distance traversed by the MIMU between two consecutive ICs of the same foot. To estimate it, the proper acceleration signals were first expressed in the GCS, then gravity was removed. For each gait cycle analyzed, a specific motor task coordinate system (MTCS) was defined [55]; the vertical axis (V) was made to coincide with the gravity direction whereas the anterior-posterior (AP) axis was made to coincide with the direction of progression, which was determined as the direction of maximum average velocity obtained by integrating the horizontal acceleration components using the Optimally Filtered Direct and Reverse Integration (OFDRI) technique [56], while the ML axis was defined as the direction orthogonal to the AP axis. The latter MTCS has the advantage to do not be affected by errors in the heading estimates [46]. For each gait cycle, the AP acceleration component expressed in the MTCS was integrated using the OFDRI [37] from the 40% of the stance phase when at least a selected point of the foot (the calcaneous) can be considered fixed with respect to the ground [47]. The OFDRI technique requires the knowledge of the final value of the integral to set a cut off frequency for the high pass filter employed to reduce the effect of the drift in the accelerometer signals. The resulting cut-off frequency was then applied for filtering the acceleration signals in the MTCS, one gait cycle at a time. The initial integration value for the linear AP velocity of the MIMU was determined as the product of ML angular velocity and the MIMU distance from the calcaneous. The velocity values found for the final instant of the gait cycle were used as initial velocity values for the integration of the following gait cycle. Finally, the stride length was obtained as the AP displacement resulting from a further simple integration of the AP velocity previously obtained. Both temporal and spatial parameters were estimated for left and right sides.

A flowchart describing the algorithm used for estimating the spatio-temporal parameters of gait is reported in (Additional file 1).

### Statistical analysis

#### Spatio-temporal parameters estimation errors

For each gait cycle, the difference between the estimated gait parameter (IC*,* FC*,* stride, step, stance, swing durations and stride length) and the reference value provided by the gold standard (instrumented mat) was determined and referred to as the error (*e*). Its absolute value and the relevant percent value were also computed.

For each subject, descriptive statistics for the error (mean and standard deviation values) and for the absolute and percent errors (mean values) were determined for both left and right feet. A Wilcoxon signed rank test was also performed to reveal differences between the absolute errors values obtained for the affected and unaffected side at both comfortable and higher speed in the H subjects. For each subject, left and right errors were then averaged. The resulting group averages were finally computed (*me, sde, mae, %mae*).

#### Comparison of errors between comfortable and higher walking speed for each group

Given the limited sample size of the four groups, a five number summary statistics (i.e. the minimum, the maximum, the median, the first quartile and the third quartile) was used to represent the errors in estimating each gait parameter for each subject group and for both the comfortable and higher walking speed conditions. A Wilcoxon signed rank test was used to compare each subject’s mean values of the absolute errors obtained for the two walking conditions to evaluate if there were statistical differences between them. Differences were considered significant if the p-value was less than 0.05.

#### Comparison of errors between healthy elderly and pathologic groups

A Wilcoxon rank sum test was performed between the subject mean values of the absolute errors obtained for the E group and those obtained for each of the pathologic groups. Differences were considered significant if the p-value was less than 0.05.

## Results

Over 4,514 gait cycles were obtained with the instrumented mat and used for the comparative analysis. The total number of gait cycles analyzed for each subject group at the two different gait speeds along with the mean (*me*) and standard deviation (*sde*) values of the analyzed spatio-temporal parameters (gait velocity, stride time, step time, stance time, swing time and stride length) for both walking speed conditions as determined by the instrumented mat are reported in Table 2.

**Table 2:** **Number of gait cycles and mean** ***(sd)*** **of gait velocities, stride time, step time, stance time, swing time and stride length for all groups (healthy elderly – E, hemiparetic – H, Parkinson’s disease – P and choreic – C) at both comfortable (V1) and higher (V2) speed**

Group	Comfortable speed							Higher speed						
	(V1)							(V2)						
	Gait cycles	Gait velocity [m/s]	Stride time [s]	Step time [s]	Stance time [s]	Swing time [s]	Stride length [m]	Gait cycles	Gait velocity [m/s]	Stride time [s]	Step time [s]	Stance time [s]	Swing time [s]	Stride length [m]
**E**	578	1.17 (*0.16*)	1.05 *(0.10)*	0.53 *(0.05)*	0.68 *(0.07)*	0.38 *(0.03)*	1.23 *(0.15)*	610	1.49 (*0.22*)	0.92 *(0.10)*	0.46 *(0.05)*	0.58 *(0.08)*	0.34 *(0.02)*	1.35 *(0.19)*
**H**	576	0.61 (*0.24*)	1.35 *(0.24)*	0.67 *(0.12)*	0.94 *(0.17)*	0.41 *(0.10)*	0. 81 *(0.30)*	516	0.79 (*0.30*)	1.22 *(0.21)*	0.60 *(0.10)*	0.83 *(0.17)*	0.39 *(0.07)*	0.86 *(0.30)*
**P**	532	0.85 (*0.14*)	1.14 *(0.09)*	0.57 *(0.05)*	0.76 *(0.07)*	0.38 *(0.03)*	0.97 *(0.15)*	560	1.02 (*0.14*)	1.04 *(0.10)*	0.52 *(0.05)*	0.68 *(0.07)*	0.36 *(0.04)*	1.06 *(0.15)*
**C**	567	1.08 (*0.30*)	1.11 *(0.14)*	0.56 *(0.07)*	0.71 *(0.10)*	0.40 *(0.05)*	1.16 *(0.21)*	575	1.28 (*0.26*)	1.00 *(0.11)*	0.50 *(0.05)*	0.64 *(0.08)*	0.36 *(0.03)*	1.27 *(0.23)*

### Spatio-temporal parameters estimation errors

#### Gait events and temporal parameters

Neither missed nor extra GEs generated by the proposed method were observed. Therefore, all 4,514 gait cycles obtained with the instrumented mat were used for the analysis. The values of *me, sde, mae* and *%mae* of each group at both walking speeds, are presented in Table 3 for IC, FC, stride time, step time, stance time and swing time.

**Table 3:** **Values for the group mean errors (** ***me*** **), mean standard deviation of the subject errors (** ***sde*** **), mean absolute errors (** ***mae*** **) and the percent of it (** ***%mae*** **) in estimating gait events (IC and FC) and temporal parameters (stride, step, stance and swing time) for the four groups (healthy elderly – E, hemiparetic – H, Parkinson’s disease – P and choreic – C)**

***p***	Group	***me***(***sde***) [ms]		***mae***[ms]		***%mae***	
		V1	V2	V1	V2	V1	V2
**IC**	**E**	2 (10)	9 (10)	10	12	-	-
	**H**	0 (17)	3 (15)	17	15	-	-
	**P**	11 (11)	22 (9)	15	22	-	-
	**C**	7 (13)	7 (11)	12	13	-	-
**FC**	**E**	7 (15)	16 (9)	20	19	-	-
	**H**	11 (18)	13 (17)	21	21	-	-
	**P**	5 (18)	0 (15)	22	19	-	-
	**C**	2 (14)	6 (13)	18	16	-	-
**Stride time**	**E**	0 (14)	0 (13)	10	10	*1%*	*1%*
	**H**	0 (17)	0 (16)	13	12	*1%*	*1%*
	**P**	1 (15)	0 (13)	12	10	*1%*	*1%*
	**C**	0 (17)	0 (15)	13	12	*1%*	*1%*
**Step time**	**E**	0 (15)	0 (14)	12	12	*2%*	*3%*
	**H**	1 (26)	0 (22)	22	22	*3%*	*4%*
	**P**	0 (15)	0 (14)	12	11	*2%*	*2%*
	**C**	0 (18)	0 (16)	14	13	*3%*	*3%*
**Stance time**	**E**	10 (19)	25 (13)	22	28	*3%*	*5%*
	**H**	11 (11)	11 (22)	25	25	*3%*	*3%*
	**P**	15 (20)	21 (18)	26	27	*3%*	*4%*
	**C**	5 (18)	1 (17)	22	19	*3%*	*3%*
**Swing time**	**E**	9 (19)	25 (13)	22	27	*6%*	*8%*
	**H**	11 (23)	10 (22)	25	25	*6%*	*6%*
	**P**	16 (21)	21 (18)	24	27	*7%*	*8%*
	**C**	5 (19)	0 (17)	22	19	*6%*	*5%*

#### Gait spatial parameters

The *me, sde, mae, %mae* of the stride length are presented in Table 4 for each group and at both walking speeds.

**Table 4:** **Values for the group mean error (** ***me*** **), mean standard deviation of the subject error (** ***sde*** **), mean absolute error (** ***mae*** **) and the percent of it (** ***%mae*** **) in estimating stride length for the four (healthy elderly – E, hemiparetic – H, Parkinson’s disease – P and choreic – C)**

Group	***me***(***sde***) [mm]		***mae***[mm]		***%mae***	
	V1	V2	V1	V2	V1	V2
**E**	2 (19)	0 (19)	18	15	*1%*	*1%*
**H**	−6 (27)	−11 (22)	21	20	*3%*	*3%*
**P**	4 (21)	1 (19)	18	16	*2%*	*2%*
**C**	−8 (29)	−7 (31)	26	24	*2%*	*2%*

The agreement in estimating gait spatio-temporal parameters between the proposed MIMU based approach and the reference method is also reported using Bland-Altman plots (see Additional file 2).

No statistically significant differences were found for all the gait parameters at both comfortable and higher speed between the subject mean values of the absolute errors obtained for the affected and unaffected side of H subjects.

### Comparison of errors between comfortable and higher walking speed for each group

**Figure 3:**
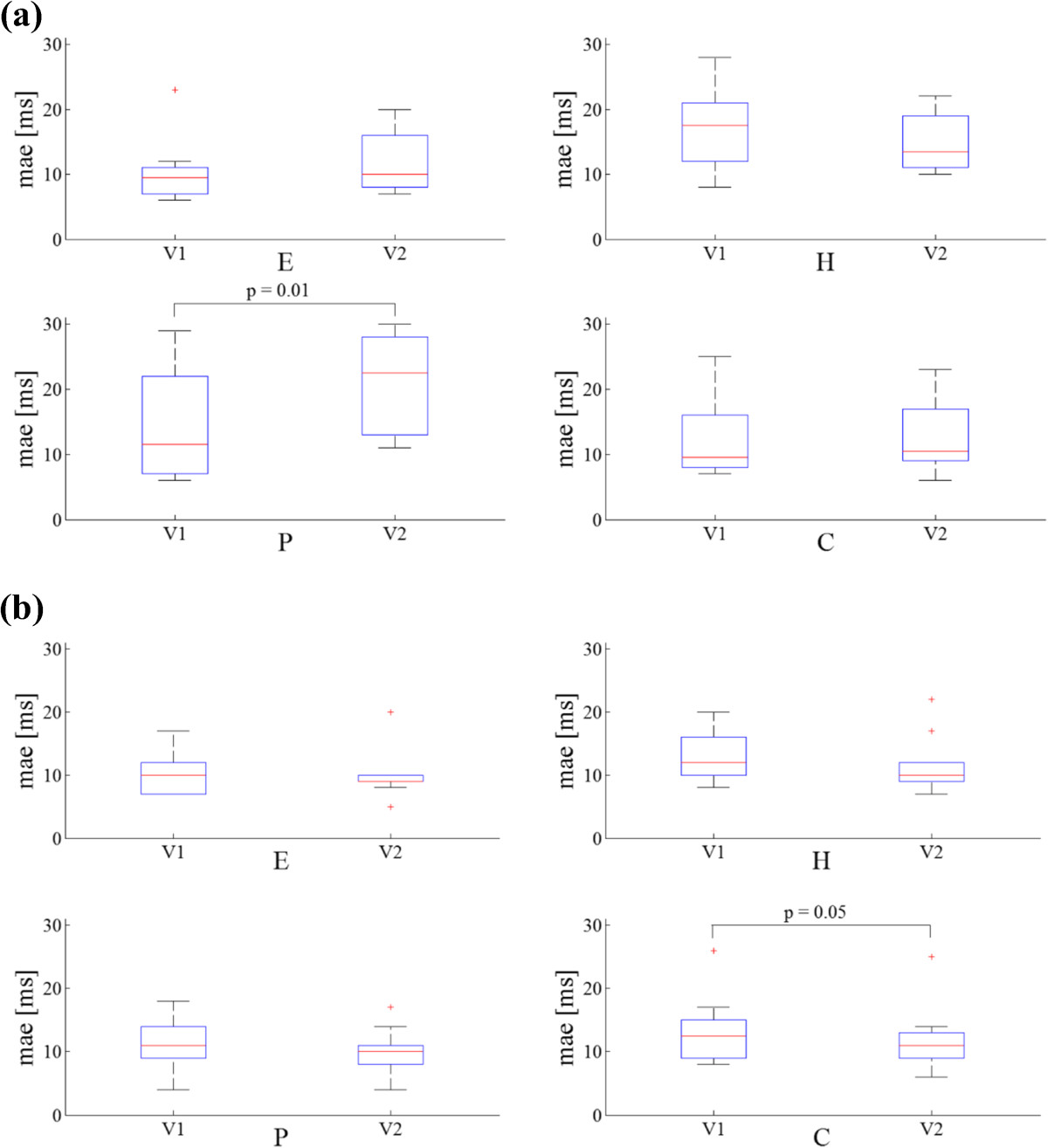
**Minimum, first quartile (q1), median, third quartile (q3) and maximum values of mean absolute errors (** ***mae*** **) relative to: (a) IC and (b) stride time for all groups (healthy elderly – E, hemiparetic – H, Parkinson’s disease – P and choreic – C) and for both comfortable (V1) and higher (V2) speed.** Errors larger than q_1_ + 1.5(q_3_ + q_1_) or smaller than q_1_–1.5(q_3_–q_1_) are considered outliers and represented with red marks (+).

All *mae* values were not significantly different between walking speeds except for the *mae* of the IC of the P group. The stride time *mae* estimated for the C group was borderline statistically significant (p = 0.05). In Figure 3 the five-number summary plots for the above mentioned parameters are reported.

### Comparison of errors between healthy elderly and pathologic groups

**Figure 4:**
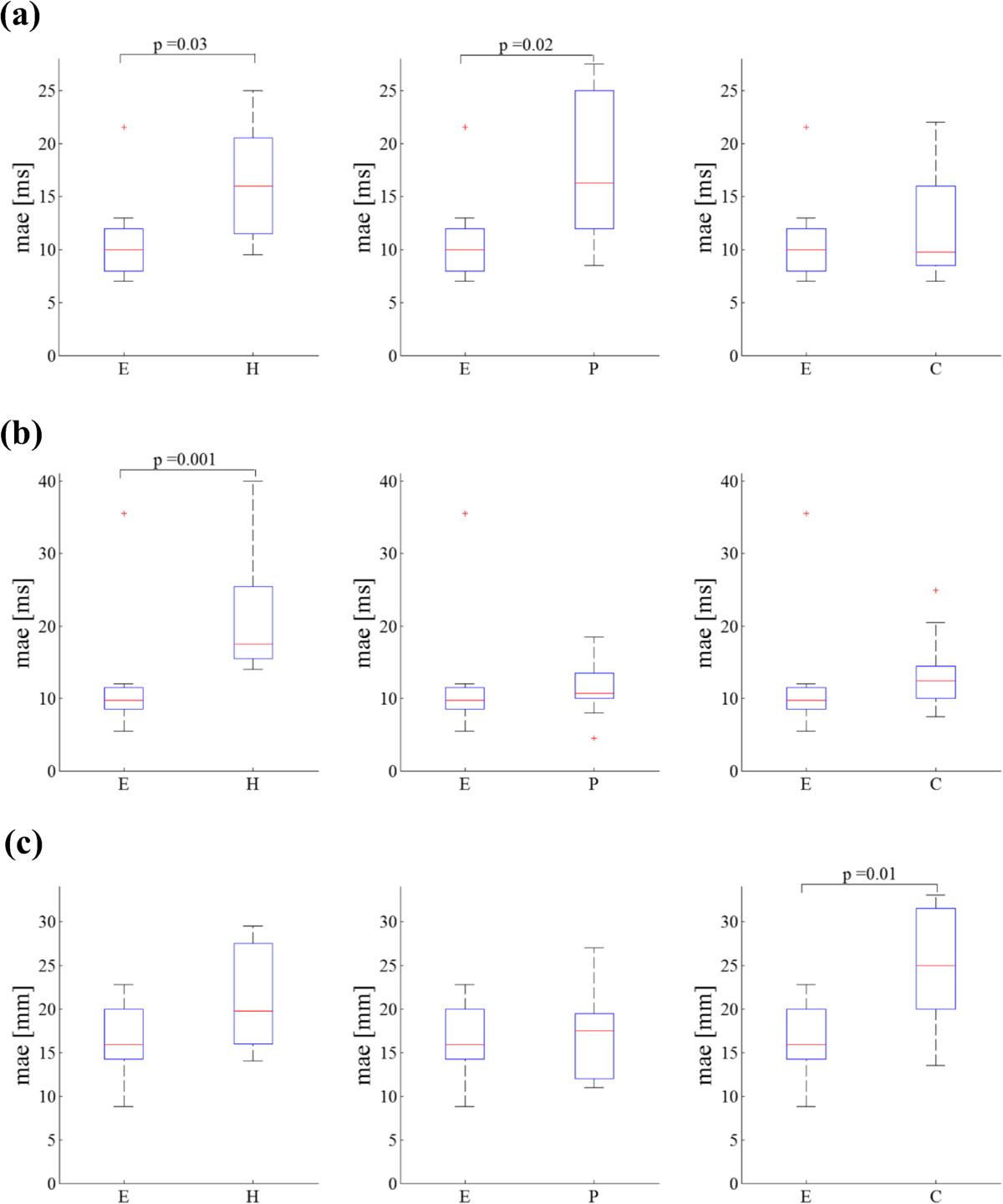
**Minimum, first quartile (q1), median, third quartile (q3) and maximum values of mean absolute errors (mae) relative to: (a) IC, (b) step time and (c) stride length for all groups (healthy elderly – E, hemiparetic – H, Parkinson’s disease – P and choreic – C).** Errors larger than q_1_ + 1.5(q_3_ + q_1_) or smaller than q_1_–1.5(q_3_–q_1_) are considered outliers and represented with red marks (+).

None of the *mae* were significantly different between elderly and any of the pathologic groups except for the IC of the E and the P groups as well as that of the E and the H groups. The step time *mae* of the H group and the stride length *mae* of the C group were significantly different from those of the E group. In Figure 4, the five-numbers plots for the above mentioned parameters are reported.

## Discussion

In this study, we proposed a methodology based on the use of two magneto-inertial units attached above the ankles for the bilateral estimation of gait spatio-temporal parameters. The method exploits some invariant kinematic constraints characterizing both healthy and compromised gait to reduce the time intervals in which the initial and final contacts are sought. The method also includes an optimal integration technique to reduce the errors caused by the drift affecting the acceleration signals.

In this study we also validated the method on the gait of healthy (elderly) and pathological groups (hemiparetic, Parkinson’s disease and choreic). No missed or extra GEs were detected for any of the groups. For the elderly, hemiparetic and choreic groups, the error in identifying IC at comfortable speed were the lowest errors ever reported in the literature. For the Parkinson disease group, the average error was slightly higher than that found in one study [27] (11 ms vs. 8.7 ms), although the authors reported some false positive events.

Similarly, in detecting the FC timing, our method outperformed most of those found in the literature. For the elderly and Parkinson’s disease groups, the errors were larger (2–3 ms) than those obtained by [27]. As far as we know, no study in the literature showed lower errors than those found for the hemiparetic and choreic groups.

The stride and step time estimations exhibited, for all groups, higher accuracy than that found in any previously published method.

Stance and swing time estimation errors were one order of magnitude larger than those found for the stride time. The error found for the stance time estimate of the elderly group was larger only than that found by [29] (about 9 ms), although they did not report standard deviation values. When the method was applied to the Parkinson’s disease group, the error affecting the stance time estimate was larger only than that found in [27] (11 ms vs. 5.9 ms), but with a much lower standard deviation (11 ms vs. 29.6) at comfortable speed. No previous studies reporting stance time estimation errors in choreic and hemiplegic populations were found in the literature.

Swing time determination errors could be compared only to those obtained for healthy elderly subjects by [29], which were higher than those we found (16.5 vs. 9 ms) at comfortable speed.

For the elderly group, stride length estimation errors were negligible and comparable to those found in [49]. The errors found for all pathological groups were about one order of magnitude lower than those reported in [27, 32].

A thorough comparison of the performance of the different methods published so far could not be performed since most of the existing studies did not provide the mean absolute error which provides a better picture of the extent of estimation errors than the mean error.

As opposed to other methods [14], the present method is not influenced by walking speeds.

In conclusion, the proposed method appeared to be extremely robust since: a) it did not present neither missed nor extra GEs; b) gait speed did not substantially affect the performance of the method. Moreover, the gait spatio-temporal parameters estimates showed a similar level of precision and accuracy for both healthy and various pathologic gait patterns. The combination of robustness, precision and accuracy and makes the proposed method suitable for a routine clinical use.

As expected, the stride length estimation error was larger for the C group, most probably due to the intrinsic difficulties associated with the determination of the direction of progression from the choreic gait patterns characterized by jerky lower limb movements.

Some aspects of the proposed method may be further improved. The proposed method performs well when applied to straight line walking, however, the results cannot be extended to the analysis of gait including turns. The ZUPT was applied at 40% of the stance phase, which was shown to be the most appropriate instant when analyzing normal gait. However, there are not indications that the latter assumption is optimal for any the pathologic groups examined in this study.

## Electronic supplementary material

Additional file 1: **Flowchart of the algorithm.** Flowchart detailing operations of the gait spatio-temporal parameters estimation algorithm. (PDF 86 KB)

Additional file 2: **Bland-Altman plots.** Bland-Altman plots illustrating the agreement between selected gait spatio-temporal parameters (stride time, step time, stance time, stride length) obtained using the proposed MIMU-based method and those derived from the reference method for each subjects group. Limits of agreement are specified as average difference (solid line) ±1.96 standard deviation of the difference (dotted line). Data from normal and fast walking conditions are merged for each subjects group. (PDF 232 KB)
