# Supplementary figures and images for: Estimation of step-by-step spatio-temporal parameters of normal and impaired gait using shank-mounted magneto-inertial sensors: application to elderly, hemiparetic, parkinsonian and choreic gait
Source: J Neuroeng Rehabil. 2014 Nov 11;11:152. doi: 10.1186/1743-0003-11-152 (PMC4242591; doi:10.1186/1743-0003-11-152)

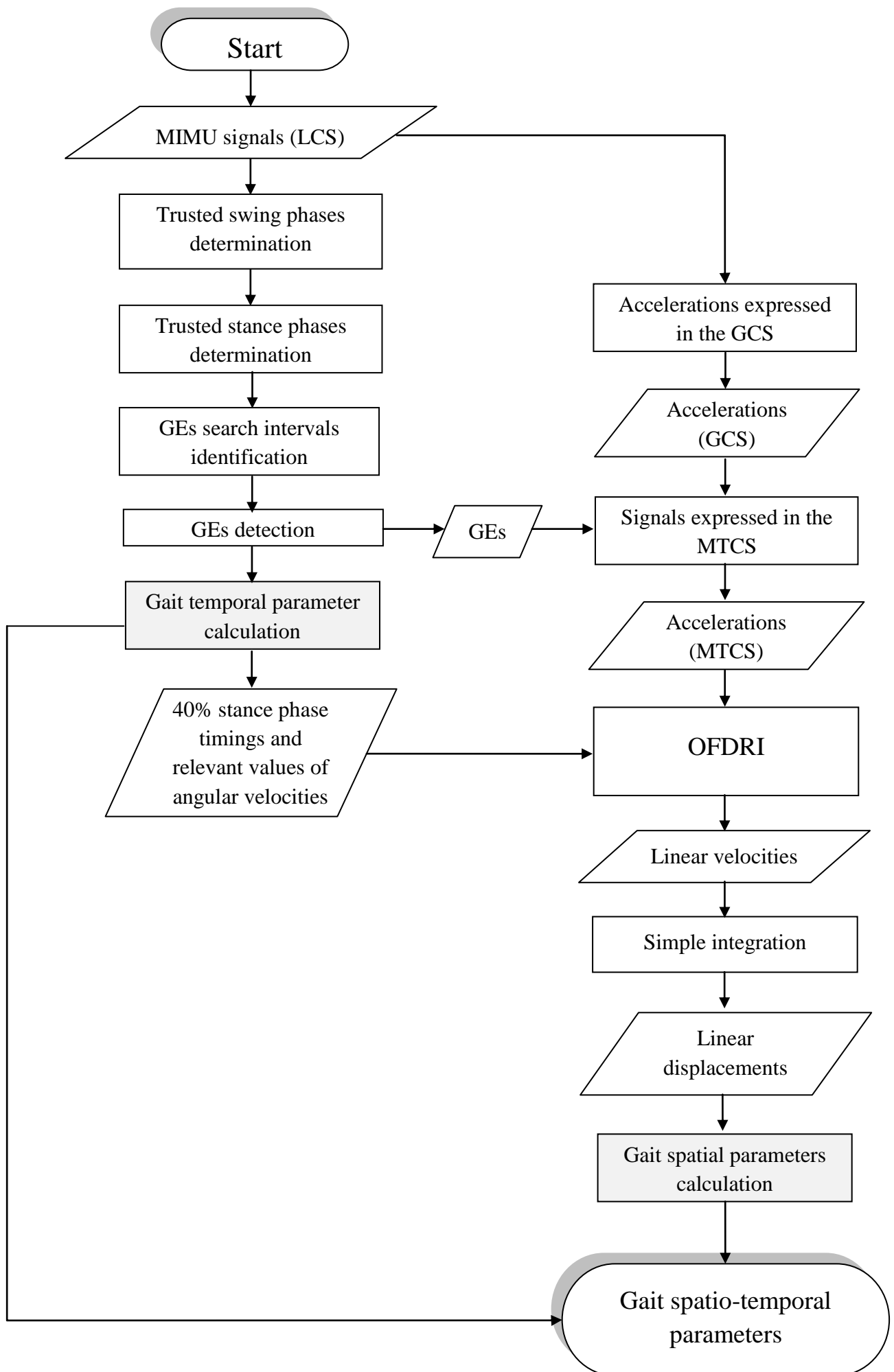

Supplement: Supplementary file 1 — Additional file 1: Flowchart of the algorithm. Flowchart detailing operations of the gait spatio-temporal parameters estimation algorithm. (PDF 86 KB) [file 12984_2014_670_MOESM1_ESM.pdf]

## STRIDE TIME (s)

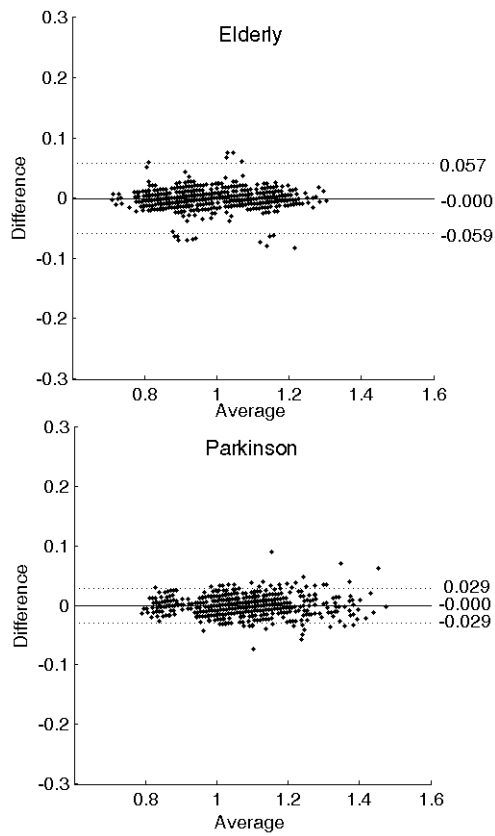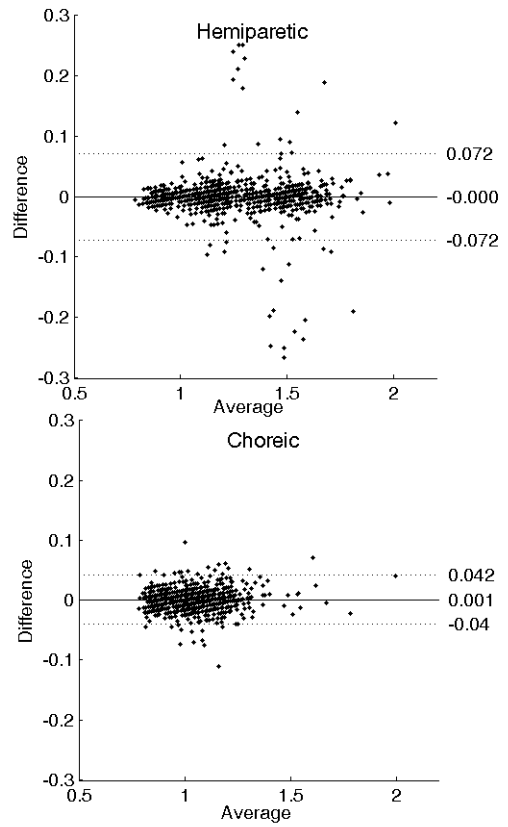

## STEP TIME (s)

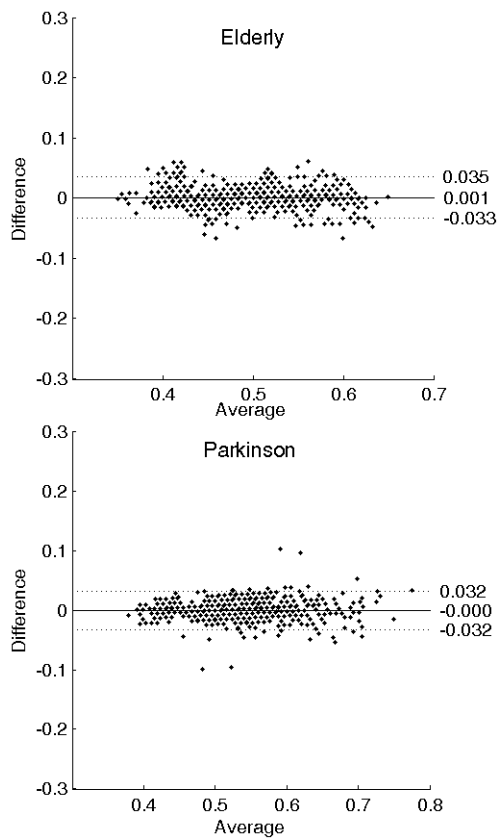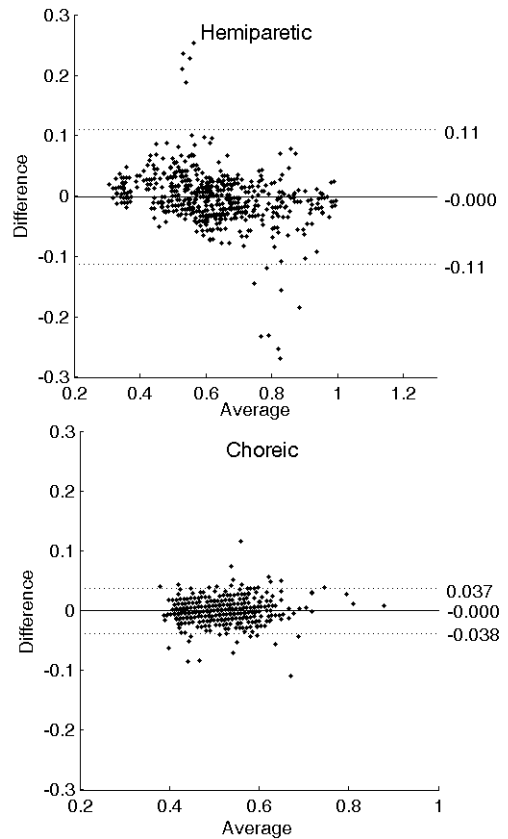

## STANCE TIME (s)

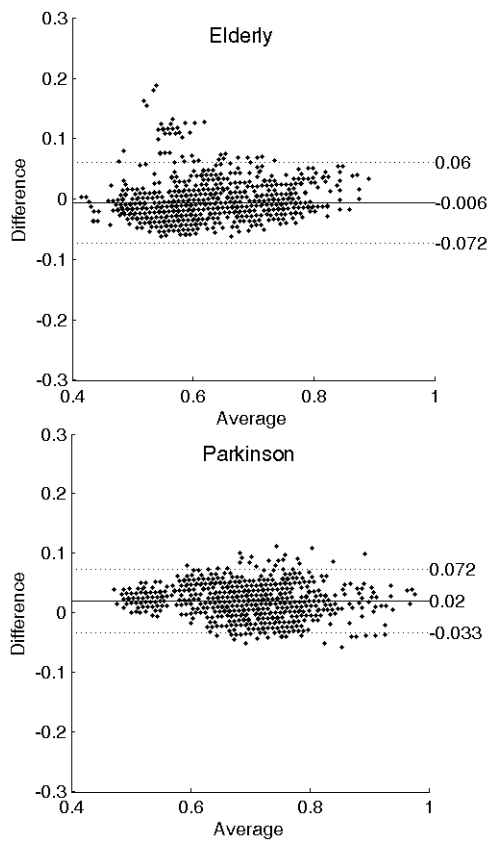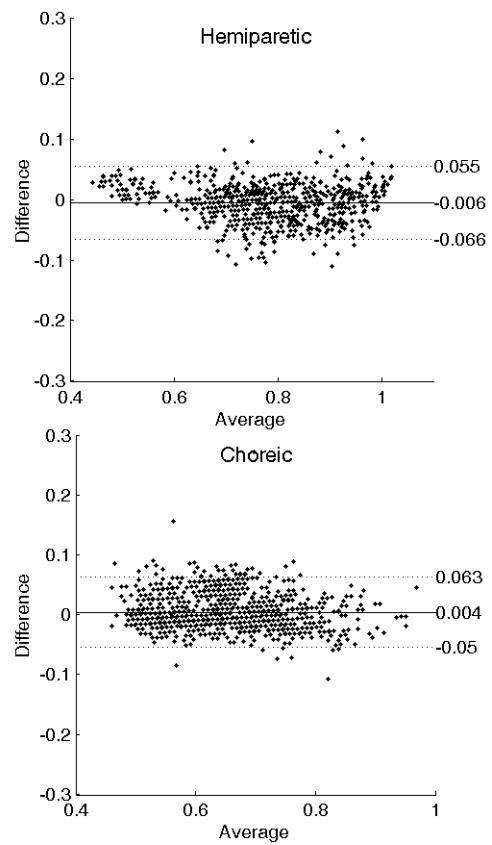

## STRIDE LENGTH (m)

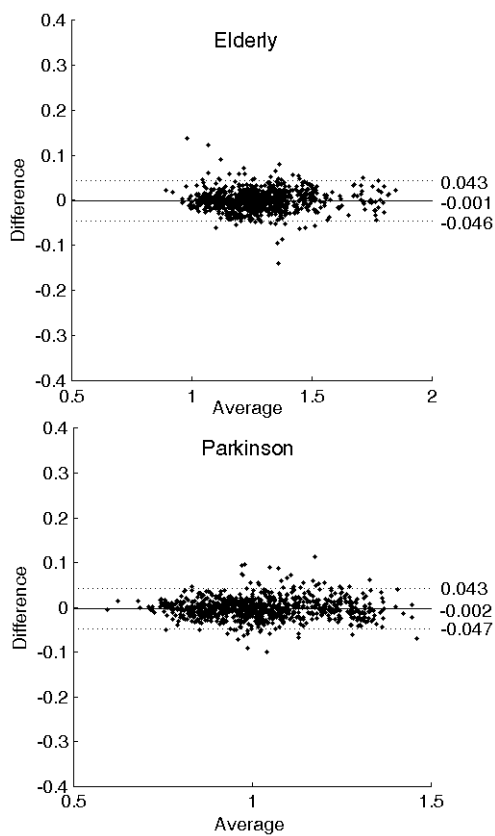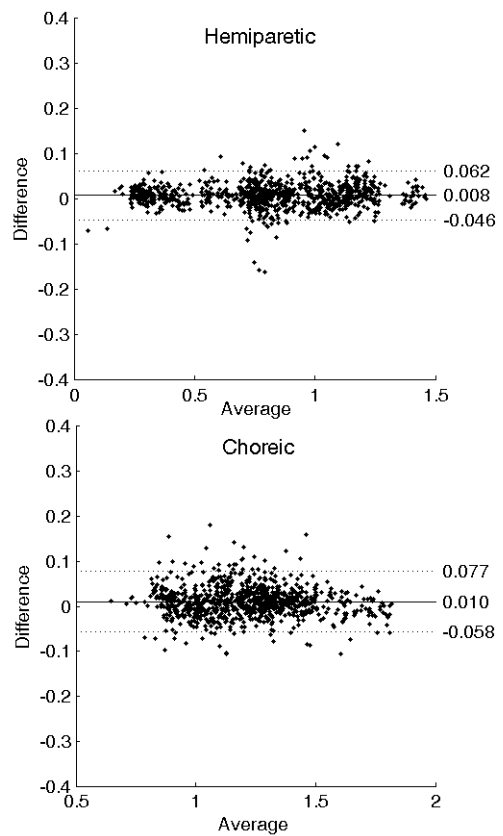

Supplement: Supplementary file 2 — Additional file 2: Bland-Altman plots. Bland-Altman plots illustrating the agreement between selected gait spatio-temporal parameters (stride time, step time, stance time, stride length) obtained using the proposed MIMU-based method and those derived from the reference method for each subjects group. Limits of agreement are specified as average difference (solid line) ±1.96 standard deviation of the difference (dotted line). Data from normal and fast walking conditions are merged for each subjects group. (PDF 232 KB) [file 12984_2014_670_MOESM2_ESM.pdf]
